# Dimensional changes of alveolar bone after orthodontic expansion with Invisalign^®^ aligners: study by Cone Beam Computed Tomography

**DOI:** 10.1590/2177-6709.30.5.e2525225.oar

**Published:** 2026-01-09

**Authors:** Bruno Boaventura VIEIRA, Guido Artemio MARAÑÓN-VÁSQUEZ, Marcio Antonio de FIGUEIREDO, Maria Bernadete Sasso STUANI, Fábio Lourenço ROMANO, Mirian Aiko Nakane MATSUMOTO

**Affiliations:** 1 University of São Paulo, Ribeirão Preto School of Dentistry, Department of Pediatric Dentistry (Ribeirão Preto, São Paulo, Brazil).; 2 Private Practice (Sorocaba, São Paulo, Brazil).

**Keywords:** Alveolar bone loss, Clear aligners, Cone-beam computed tomography, Periodontics, Orthodontics, Perda do osso alveolar, Alinhadores transparentes, Tomografia computadorizada de feixe cônico, Periodontia, Ortodontia

## Abstract

**Objective:**

This study aimed to evaluate changes, using Cone-Beam Computed Tomography (CBCT), in the buccal and palatal alveolar bone of maxillary premolars in young adult individuals who underwent dentoalveolar expansion with Invisalign^®^ aligners, and to compare these changes across different facial types.

**Methods:**

Forty-five patients (32 women and 13 men; mean age: 34.2 years) underwent orthodontic expansion with clear aligners. CBCT scans were obtained before (T0) and after expansion of the maxillary arch (T1), with a mean treatment period of 21.4 months. Changes in alveolar bone height and thickness were assessed at the cervical, middle, and apical regions of the maxillary premolars across different facial types (mesofacial, brachyfacial, and dolichofacial). The level of significance was set at 5%.

**Results:**

Regarding bone thickness, significant differences between T0 and T1 were observed for tooth 15 and 24 in the mesofacial group; tooth 25 in the dolichofacial group; and teeth 14, 15, 24, and 25 in the brachyfacial group. Regarding bone height, a significant difference was found for tooth 15 in the brachyfacial group. No differences were observed in bone height or thickness when comparing the different facial types.

**Conclusion:**

Minor alveolar bone changes in height and/or thickness of maxillary premolars were observed across all facial types between the evaluated time points. No significant differences in alveolar bone alterations were found among the facial types; however, when analyzed separately, greater changes in alveolar thickness and height were observed in the horizontal growth group.

## RESUMO

**Objetivo:**

Avaliar as alterações, por meio de Tomografia Computadorizada de Feixe Cônico (TCFC), no osso alveolar vestibular e palatino de pré-molares superiores em indivíduos adultos jovens que foram submetidos à expansão dentoalveolar com alinhadores Invisalign® e compará-las nos diferentes tipos faciais.

**Método:**

Quarenta e cinco pacientes (32 mulheres e 13 homens, média de 34,2 anos) realizaram expansão ortodôntica com alinhadores dentários. TCFCs foram realizadas antes (T0) e após expansão do arco superior (T1), período médio de tratamento de 21,4 meses. Foram avaliadas as alterações ósseas alveolares, em altura e espessura, das regiões cervical, média e apical de pré-molares superiores nos diferentes tipos faciais (mesofacial, braquifacial e dolicofacial). O nível de significância adotado foi de 5%.

**Resultados:**

Em relação à espessura óssea, houve diferença significante entre os tempos T0 - T1 para os dente 15 e 24 do grupo mesofacial; dente 25 do grupo dolicofacial e dentes 14, 15, 24 e 25 para o grupo braquifacial. Em relação à altura óssea, houve diferença para o dente 15 do grupo braquifacial. Não houve diferença em nenhuma das medidas de altura e espessura óssea comparando-se os diferentes tipos faciais.

**Conclusão:**

Verificaram-se pequenas alterações ósseas alveolares, em altura e/ou espessura, de pré-molares superiores para todos os tipos faciais entre os tempos avaliados. Não foram observadas diferenças nas alterações ósseas alveolares entre os tipos faciais; porém, quando analisados separadamente, foram observadas maiores alterações na espessura e altura alveolares no grupo de crescimento horizontal.

## INTRODUCTION

Orthodontic treatments are based on induced tooth movement, which triggers a series of physiological processes occurring in the supporting periodontal structures of the tooth. If the patient’s biological limits are not respected during the therapeutic approach, the alveolar bone plates may be compromised, increasing the likelihood of developing dehiscences and/or fenestrations.^1^ ^-^ ^3^

The literature has shown that dentoalveolar expansion is one of the movements that most compromises the buccal alveolar bone.⁴ CBCT evaluations have demonstrated significant reductions in the thickness of the buccal bone tissue in patients who underwent arch expansion with different types of orthodontic appliances.⁵

To date, despite the growing number of cases treated with clear aligners, there are few studies assessing their effects on the alveolar bone. Studies on buccal bone thickness are commonly found in the literature^4^; however, none have evaluated alveolar changes across different facial types. The lack of information regarding the alveolar effects of aligner treatment motivated us to investigate the changes that expansive movement may cause in the supporting bone of the maxillary premolars in adult patients, as well as its relationship with facial growth pattern, using three-dimensional CBCT images, which combine high definition and the absence of overlapping structures, thus allowing precise measurements of bone thickness and depth.

The aim of the study was to compare alveolar bone changes, in thickness and height, in maxillary premolars of patients with different skeletal patterns after expansive movement with aligners.

## MATERIAL AND METHODS

This study was approved by the Human Research Ethics Committee of the Ribeirão Preto School of Dentistry, University of São Paulo (#10113319.1.0000.5419).

Based on a preliminary power analysis, a minimum sample size of 28 participants was required to achieve an 80% study power with a significance level of 0.05. Adult patients over 18 years of age with full permanent dentition (except third molars), maxillary dental crowding less than 3mm, absence of anterior and posterior crossbites, without dental anomalies, with good general and oral health, without active periodontal disease, no history of using medications affecting bone metabolism in the past year and treated with clear aligners for maxillary dentoalveolar expansion were considered eligible. Forty-five white patients, 32 females and 13 males, aged between 18 and 45 years, with a mean age of 34.2 years were included in this retrospective study. The patients were classified according to the Ricketts’ mandibular plane angle^6^ in mesofacial (26° ± 4°), dolichofacial (>30°), and brachyfacial patients (<22°). Characteristics of the patients are showed in Table 1.

**Table 1:** Sample characteristics.

Variables	Mandibular growth pattern			Total
	Mesofacial	Dolichofacial	Brachyfacial	
Gender - n (%)				
Male	3 (20)	2 (13)	8 (53)	13 (29)
Female	12 (80)	13 (87)	7 (47)	32 (71)
Age (years) - mean ± SD	33.3 ± 8.0	36.5 ± 9.7	32.7 ±9.2	34.2 ± 8.9
FH/GoMe- mean ± SD	25.5 ± 2.2	33.7 ± 3.4	16.6 ± 2.1	25.3 ± 7.7
Treatment time (months) - mean ± SD	25.3 ± 10.0	19.8 ± 10.0	19.1 ± 7.6	21.4 ± 9.5

FH/GoMe = Ricketts’ mandibular plane.

All patients were treated with clear aligners from the Invisalign^®^ system (Align Technology, San Jose, CA, USA) by the same orthodontist (MF) in a private clinic. Composite resin attachments were used in all cases and placed according to the needs determined in the virtual treatment plan (ClinCheck). Patients changed their aligners every one to two weeks, with the interval individually determined based on the discomfort each patient reported during the switching days. Interproximal enamel reduction (IPR) in the upper arch was performed in 70% of the sample to resolve dental crowding and, when necessary, to minimize the black triangle space between the incisors. Oral hygiene was monitored at each follow-up appointment. The average treatment duration was 21.4 months.

Pre and post-treatment CBCT scans were used to measure dentoalveolar expansion and the amount of alveolar bone tissue present on the buccal and palatal surfaces of the maxillary first and second premolars. The CBCT images were obtained with the i-CAT Classic 3D Dental Imaging System (Imaging Sciences, Hatfield, PA, USA), at 120 kVp, 8 mA, 40-second scan time, with a voxel size of 0.25mm. Data from each patient were saved in DICOM format. OnDemand3D software (Cybermed Inc., Yuseong-gu, Daejeon, South Korea) was used to obtain linear measurements using the “ruler” tool.

**Figure 1:**
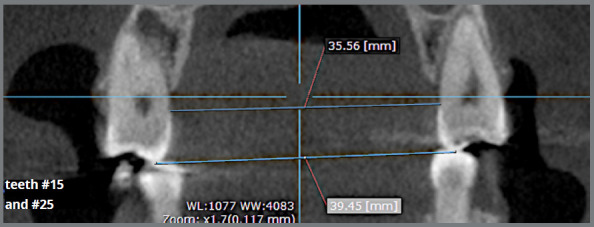
Reference points for assessing dentoalveolar expansion.

Dentoalveolar expansion in the premolar region was confirmed by means of transverse measurements using the cervical regions and the tips of the palatal cusps of the maxillary premolars as reference points (Fig. 1).

**Figure 2:**
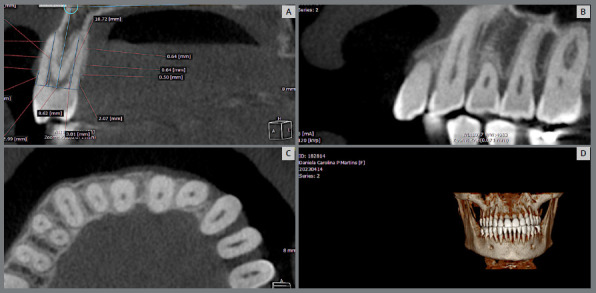
A) OnDemand 3D software. B) Coronal view. C) Axial view. D) Three-dimensional model

The alveolar bone of the premolars on the palatal and buccal surfaces was evaluated using the following methodology: the dental roots were evaluated throughout their entire length to determine the region with the greatest volume of bone tissue. Vertical and horizontal lines were automatically adjusted by the software according to the long axis of the tooth. Once the image positioning was defined, the coronal view was used to perform the measurements (Fig. 2). The measurements followed the protocol established by Nahás-Scocate and Scocate.^7^

## ALVEOLAR MEASUREMENTS

To measure the alveolar bone tissue of the maxillary first and second premolars, two reference lines were created from the tips of the buccal and palatal cusps, following the long axis of the buccal and palatal roots. A line was drawn through the buccal and palatal cemento-enamel junctions (CEJ), and the root length (CEJ-apex) was then measured and divided into three thirds.

The cervical third was measured in two ways: the distance from the CEJ to the alveolar bone crest buccal and palatal (CEJ-BC and CEJ-PC); and the thickness of the alveolar bone crest at a distance of 3mm from the CEJ in the apical direction (CBT and CPT).

**Figure 3:**
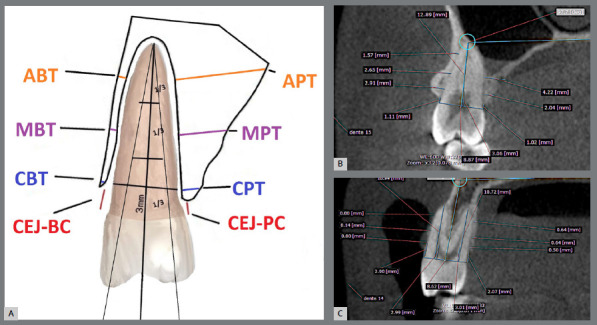
A) Alveolar measurements: CEJ-BC = distance from the cementoenamel junction to the buccal bone crest; CBT (cervical buccal thickness) = bone tissue present on the buccal surface (cervical region) at a distance of 3.0 mm from the CEJ; MBT (middle buccal thickness) = bone tissue present at the midpoint of the middle third of the buccal surface; ABT (apical buccal thickness) = bone tissue present at the midpoint of the apical third of the buccal surface; CEJ-PC = distance from the cementoenamel junction to the palatal bone crest; CPT (cervical palatal thickness) = bone tissue present on the palatal surface (cervical region) at a distance of 3.0 mm from the CEJ; MPT (middle palatal thickness) = bone tissue present at the midpoint of the middle third of the palatal surface; APT (apical palatal thickness) = bone tissue present at the midpoint of the apical third of the palatal surface. B, C) Measurement of bone plate thickness and height in the premolar region: two reference lines, one originating from the buccal cusp and the other from the palatal cusp. Bone plate measurements were taken perpendicularly to the long axis of the root on both the buccal and palatal surfaces.

The middle and apical thirds were further divided in half, thereby obtaining midpoints for the middle (MBT and MPT) and apical thirds (ABT and APT) of the roots, where bone tissue thickness was measured (Fig. 3).

All measurements were performed on the buccal and palatal surfaces, positioning the “ruler” tool from the outer boundary of the cortical bone to its inner boundary,, corresponding to buccal bone thickness (CBT, MBT, ABT). The same procedure was performed to obtain palatal bone thickness at the cervical, middle, and apical thirds (CPT, MPT, APT) for each tooth (Fig. 3).

Measurements in 15 CBCT were repeated after an interval of at least two weeks. The intraclass correlation test (ICC), Dahlberg’s formula, and the Bland-Altman method were used to assess method error. The measurements were shown to be reliable (ICC > 0.7), reasonably precise, and no evidence of systematic error was observed.

## STATISTICAL ANALYSIS

Descriptive statistics were used to report the data from the tomographic measurements (means and standard deviations). Paired t-tests were used to compare pre- and post-treatment alveolar measurements within each group. One-way ANOVA was used to compare differences in alveolar measurements between groups according to mandibular growth pattern. Test assumptions were evaluated using the Shapiro-Wilk normality test and the Levene test for homogeneity of variances. All analyses were performed using Jamovi 2.3.21, adopting a significance level of 5%.

## RESULTS

There was significant dentoalveolar expansion between the evaluated moments for first and second premolars, both for the cervical region and for the tips of the palatine cusps (Table 2).

**Table 2:** Evaluation of dentoalveolar expansion between time points.

Outcomes (mean ± SD)	T0	T1	T0 - T1	P valor
First premolars (cerv) (mm)	27.04 ± 2.03	28.68 ± 1.41	1.64 ± 1.31	<0.001*
First premolars (cusp) (mm)	30.40 ± 2.17	33.00 ± 1.55	2.60 ± 1.70	<0.001*
Second premolars (cerv) (mm)	32.36 ± 2.97	33.82 ± 3.29	1.46 ± 2.19	0.002*
Second premolars (cusp) (mm)	34.97 ± 3.18	37.49 ± 2.21	2.52 ± 1.88	<0.001*

The results will be presented in the following sequence: thickness of the premolar bone plates (cervical, middle and apical regions) in the different facial patterns, and height of the premolar bone plates (buccal and palatal regions) in the different facial patterns and comparison between the three groups evaluated.

### ALVEOLAR BONE THICKNESS FOR THE MESOFACIAL PATTERN GROUP

For the mesofacial pattern, there was a significant difference between the evaluated time points (T0-T1) for MPT of tooth 15, with a difference of −0.4 ± 0.6 (mean ± SD) and (p = 0.026); MBT of tooth 24, with a difference of 0.3 ± 0.4 and (p = 0.046); and APT of tooth 24, with a difference of 0.6 ± 0.9 and (p = 0.043).

### ALVEOLAR BONE THICKNESS FOR THE DOLICHOFACIAL PATTERN GROUP

For the dolichofacial pattern, there was a significant difference between the evaluated time points (T0-T1) for MBT of tooth 25, with a difference of 0.3 ± 0.4 (mean ± SD) and (p = 0.005). There was no significant difference between time points for any tooth in relation to palatal measurements.

### ALVEOLAR BONE THICKNESS FOR THE BRACHYFACIAL PATTERN GROUP

For the brachyfacial pattern, there was a significant difference between the evaluated time points (T0-T1) for MBT of tooth 14, with a difference of 0.3 ± 0.5 (mean ± SD) and (p = 0.041); CBT of tooth 15, with a difference of 0.6 ± 0.6 and (p = 0.006); APT of tooth 15, with a difference of 0.8 ± 0.8 and (p = 0.013); MBT of tooth 24, with a difference of 0.3 ± 0.4 and (p = 0.008); and APT of tooth 25, with a difference of 0.4 ± 0.6 and (p = 0.047).

### ALVEOLAR BONE HEIGHT FOR THE MESOFACIAL PATTERN GROUP

For the mesofacial pattern, no significant difference was observed between the evaluated time points (T0-T1) for any tooth regarding the alveolar bone heights in the buccal and palatal regions. 

### ALVEOLAR BONE HEIGHT FOR THE DOLICHOFACIAL PATTERN GROUP

For the dolichofacial pattern, no significant difference was observed between the evaluated time points (T0-T1) for any tooth regarding the alveolar bone heights in the buccal and palatal regions.

### ALVEOLAR BONE HEIGHT FOR THE BRACHYFACIAL PATTERN GROUP

For the brachyfacial pattern, there was a significant difference between the evaluated time points (T0-T1) CEJ-BC of tooth 15, with a difference of −0.7 ± 0.9 and (p = 0.008) and CEJ-PC of tooth 15, with a difference of −0.6 ± 0.8 and (p = 0.014).

### COMPARISON OF ALVEOLAR BONE CHARACTERISTICS AMONG FACIAL TYPES

No significant difference was observed between the evaluated time points (T0-T1) for any tooth regarding buccal and palatal measurements when compared among the different facial types (Table 3).

**Table 3:** Comparison among different facial types.

T0-T1 (mean ± SD)	Mandibular growth pattern			P-valor
	Mesofacial	Dolichofacial	Brachyfacial	
CEJ-BC (mm) - tooth #14	-0.2 ± 1.0	0.7 ± 2.2	-0.1 ± 0.9	0.487
CEJ-PC (mm) - tooth #14	0.0 ± 0.8	-0.2 ± 1.4	-0.3 ± 0.8	0.612
CBT (mm) - tooth #14	0.2 ± 0.6	-0.1 ± 0.4	0.1 ± 0.6	0.291
MBT (mm) - tooth #14	0.1 ± 0.3	0.1 ± 0.4	0.3 ± 0.5	0.308
ABT (mm) - tooth #14	0.0 ± 0.3	0.0 ± 0.4	0.1 ± 0.3	0.846
CPT (mm) - tooth #14	0.1 ± 0.4	0.0 ± 0.4	0.0 ± 0.3	0.947
MPT (mm) - tooth #14	0.2 ± 0.5	0.2 ± 0.8	0.0 ± 0.4	0.397
APT (mm) - tooth #14	0.5 ± 1.3	0.5 ± 1.0	0.0 ± 0.5	0.213
Tooth/PP (degrees) - tooth #14	-4.0 ± 8.2	-3.3 ± 4.2	-3.9 ± 4.5	0.93
CEJ-BC (mm) - tooth #15	-0.1 ± 0.5	-0.2 ± 1.1	-0.7 ± 0.9	0.068
CEJ-PC (mm) - tooth #15	0.1 ± 0.7	-0.1 ± 0.9	-0.6 ± 0.8	0.07
CBT (mm) - tooth #15	0.3 ± 0.6	0.2 ± 0.6	0.6 ± 0.6	0.325
MBT (mm) - tooth #15	0.1 ± 0.6	0.3 ± 0.5	0.2 ± 0.6	0.789
ABT (mm) - tooth #15	0.0 ± 0.6	0.1 ± 0.4	0.1 ± 0.5	0.934
CPT (mm) - tooth #15	0.0 ± 0.6	0.1 ± 0.7	0.0 ± 0.5	0.927
MPT (mm) - tooth #15	-0.4 ± 0.6	-0.1 ± 0.6	0.3 ± 1.0	0.132
APT (mm) - tooth #15	0.1 ± 0.9	0.1 ± 0.7	0.8 ± 0.8	0.095
Tooth/PP (degrees) - tooth #15	-3.2 ± 4.4	-1.9 ± 6.2	-4.2 ± 4.6	0.538
CEJ-BC (mm) - tooth #24	0.0 ± 0.8	-0.2 ± 1.3	-0.1 ± 1.4	0.939
CEJ-PC (mm) - tooth #24	-0.2 ± 0.9	0.0 ± 0.7	-0.1 ± 0.6	0.707
CBT (mm) - tooth #24	0.1 ± 0.3	0.1 ± 0.4	0.2 ± 0.5	0.716
MBT (mm) - tooth #24	0.3 ± 0.4	0.2 ± 0.4	0.3 ± 0.4	0.853
ABT (mm) - tooth #24	-0.1 ± 0.3	0.0 ± 0.2	0.0 ± 0.3	0.698
CPT (mm) - tooth #24	-0.1 ± 0.4	-0.1 ± 0.4	0.0 ± 0.4	0.863
MPT (mm) - tooth #24	-0.1 ± 0.7	-0.1 ± 0.3	0.1 ± 1.0	0.784
APT (mm) - tooth #24	0.6 ± 0.9	0.0 ± 0.9	-0.2 ± 1.2	0.12
Tooth/PP (degrees) - tooth #24	-5.2 ± 5.3	-5.3 ± 3.5	-2.8 ± 5.8	0.392
CEJ-BC (mm) - tooth #25	-0.1 ± 0.6	-0.1 ± 0.6	-0.2 ± 0.7	0.839
CEJ-PC (mm) - tooth #25	-0.1 ± 0.9	-0.4 ± 0.7	0.1 ± 0.6	0.242
CBT (mm) - tooth #25	0.2 ± 0.7	0.2 ± 0.4	0.3 ± 0.7	0.893
MBT (mm) - tooth #25	0.2 ± 0.5	0.3 ± 0.4	0.2 ± 0.5	0.821
ABT (mm) - tooth #25	-0.1 ± 0.4	0.2 ± 0.3	0.1 ± 0.3	0.279
CPT (mm) - tooth #25	-0.2 ± 0.4	0.1 ± 0.5	-0.1 ± 0.4	0.305
MPT (mm) - tooth #25	-0.1 ± 0.8	0.2 ± 0.5	0.3 ± 0.7	0.364
APT (mm) - tooth #25	0.4 ± 1.1	0.3 ± 0.7	0.4 ± 0.6	0.948
Tooth/PP (degrees) - tooth #25	-6.1 ± 5.8	-4.1 ± 3.6	-5.7 ± 5.8	0.462

*Difference between time points T0 and T1 (p < 0.05).

## DISCUSSION

The effects of orthodontic dental expansion on alveolar bone height and/or thickness have been widely analyzed, especially about traditional mechanics with conventional fixed appliances.^8^ ^,^ ^9^ However, few studies have explored these characteristics with clear aligners.^4^ ^,^ ^10^ ^,^ ^11^

Due to the retrospective nature of this study, initial periodontal records, including periodontal mapping, were not available; however, patients did not present active periodontal disease and were instructed with a standardized oral hygiene protocol before and during treatment by a periodontics specialist, including scaling and root planing every three months throughout orthodontic therapy.

Allahham et al.¹¹ Evans and Berant^12^ reported high risks of orthodontic relapse and periodontal complications such as dehiscences, fenestrations, and gingival recessions following expansive treatment with Invisalign^®^ aligners. Considering the results of our research, maxillary dentoalveolar expansion with clear aligners did not cause significant changes in the alveolar bone tissue, since the dimensions of the buccal and palatal bone plates did not undergo significant changes in the majority of the patients evaluated, with only slight reductions in the alveolar bone thickness in the middle third on the buccal surface of the roots of the maxillary second premolars and an increase in dehiscence only in the cervical third of tooth 15. 

The cervical bone region should be a point of attention in expansion mechanics for adult patients, given that the buccal direction of tooth inclination moves the dental element toward the vestibular region, where the alveolar bone is thin. The literature reports that the distance from the CEJ to the alveolar bone crest (CEJ-BC) may range from 1.5 to 2 mm in healthy patients, with alveolar bone loss considered when this distance exceeds 2mm.^13^

In the present study, only the mean CEJ-PC of tooth 15 was below 2mm at the initial time point. For the other teeth, the dehiscences exceeded the 2 mm threshold (CEJ-BC = 2.3 to 2.9mm and CEJ-PC = 1.9 to 2.3mm), indicating the difficulty of maintaining alveolar bone levels considered ideal, even in adult patients without active periodontal disease. Barreda et al.⁴ found similar results, with higher pre-treatment means for first premolars (CEJ-BC = 3.05mm) and second premolars (CEJ-BC = 2.06mm).

Additionally, a significant increase was observed only in the JEC-BC measurement of tooth 15 in brachyfacial patients, demonstrating that expansive movement with aligners, combined with periodic periodontal control, requires caution, but can be safe for the patient. This appears to be related to good control of tooth movement and improved ease of oral hygiene associated with the removable nature of orthodontic aligners. Barreda et al.⁴ reported that 81.6% of first premolars and 68.4% of second premolars showed a decrease in the CEJ-BC measurement, indicating that clear aligners do not cause dehiscences in the alveolar bone tissue in the cervical region after expansion.

An important finding of this study was that despite the significant increase in maxillary arch width due to the expansive movement, the bone tissue did not undergo significant reductions, suggesting that the use of clear aligners did not adversely affect alveolar bone height or thickness.

Our study is the first to evaluate the effects of dental expansion with aligners on the alveolar bone in different facial types, as the literature recognizes differences in alveolar bone patterns between hypodivergent and hyperdivergent facial types.^14^ ^,^ ^15^ This may trigger different outcomes in the physiology of tooth movement. According to Yaseen et al.,^16^ individuals with hyperdivergent facial patterns may present greater challenges during orthodontic tooth movement due to thinner alveolar bone, which limits tooth movement.

When comparing facial types, no differences were found in alveolar bone changes after expansion with orthodontic aligners. Evaluating each group separately, greater stability was observed within the dolichofacial group, with reduction occurring only in buccal bone thickness of the middle third (MBT) of tooth 25. For the mesofacial group, significant differences were found in buccal bone thickness of the middle third (MBT), apical palatal bone thickness (APT) of tooth 24, and palatal bone thickness of the middle third (MPT) of tooth 15 - the latter being the only parameter evaluated in which bone thickness increased following expansion with aligners. However, in the horizontal growth pattern, all premolars were affected to some extent, exhibiting decreases in bone thickness, increases in bone height, or increases in dehiscences. These results may indicate that, for this facial type, special attention should be given when expansive movement is part of the treatment plan with aligner mechanics.

These findings may be explained by the fact that brachyfacial patients exhibit greater bite forces^17^ and a higher likelihood of clenching/bruxism^18^ ^,^ ^19^ which stimulates increased alveolar bone resorption.^20^ Alveolar bone morphology appears to be closely associated with facial muscle patterning and bite force, which are significantly stronger in brachyfacial individuals compared to dolichofacial individuals,^21^ which may result in different alveolar bone responses after orthodontically induced tooth movement. Saricam and Tayman^20^ suggested that mechanical loading associated with bruxism induces trabecular bone remodeling and alterations in alveolar bone density. Additionally, studies show that excessive occlusal forces can lead to resorption of cementum/alveolar bone and widening of the periodontal ligament in cervical/apical regions, regardless of periodontal health status.^22^ However, the literature remains conflicting regarding the effects of bruxism on bone structure.^23^

This study demonstrated that alveolar bone changes occur following expansive movement with clear aligners; however, further scientific evidence is needed for clinicians to apply this mechanics with greater predictability of outcomes.

## CONCLUSION

Maxillary dentoalveolar expansion with Invisalign^®^ aligners in adult patients:

1. Caused minor alveolar bone changes, in height and/or thickness, of maxillary premolars for all facial types.
2. No differences in alveolar bone changes were observed between facial types.
3. When facial types were analyzed separately, greater changes in alveolar thickness and height were observed in the brachyfacial group.
4. There were no significant differences in alveolar bone measurements following orthodontic expansion with Invisalign^®^ aligners among the different facial morphological types.
